# A high-avidity biosensor reveals plasma membrane PI(3,4)P_2_ is predominantly a class I PI3K signaling product

**DOI:** 10.1083/jcb.201809026

**Published:** 2019-03-04

**Authors:** Brady D. Goulden, Jonathan Pacheco, Allyson Dull, James P. Zewe, Alexander Deiters, Gerald R.V. Hammond

**Affiliations:** 1 Department of Cell Biology, University of Pittsburgh School of Medicine, Pittsburgh, PA; 2 Department of Chemistry, University of Pittsburgh, Pittsburgh, PA

## Abstract

Class I phosphoinositide 3-OH kinase (PI3K) signaling is central to animal growth and metabolism, and pathological disruption of this pathway affects cancer and diabetes. However, the specific spatial/temporal dynamics and signaling roles of its minor lipid messenger, phosphatidylinositol (3,4)-bisphosphate (PI(3,4)P_2_), are not well understood. This owes principally to a lack of tools to study this scarce lipid. Here we developed a high-sensitivity genetically encoded biosensor for PI(3,4)P_2_, demonstrating high selectivity and specificity of the sensor for the lipid. We show that despite clear evidence for class II PI3K in PI(3,4)P_2_-driven function, the overwhelming majority of the lipid accumulates through degradation of class I PI3K-produced PIP_3_. However, we show that PI(3,4)P_2_ is also subject to hydrolysis by the tumor suppressor lipid phosphatase PTEN. Collectively, our results show that PI(3,4)P_2_ is potentially an important driver of class I PI3K-driven signaling and provides powerful new tools to begin to resolve the biological functions of this lipid downstream of class I and II PI3K.

## Abstract (precis)

A sensitive new genetically encoded lipid biosensor for PI(3,4)P_2_ has been developed, revealing the lipid is mainly produced via PIP_3_ after activation of class I PI3K signaling. The PI(3,4)P_2_ is also a direct substrate for the tumor suppressor lipid phosphatase PTEN.

## Introduction

Class I phosphoinositide 3-OH kinase (PI3K) signaling is central to the control of growth and metabolism in animals (Vanhaesebroeck et al., 2012). Overactivation of this pathway is the most common event in cancer (Fruman et al., 2017), yet given its major role in insulin signaling, inhibition of the pathway triggers insulin resistance and type 2 diabetes (Hopkins et al., 2018). Therefore, the ability to selectively manipulate PI3K signaling could have tremendous therapeutic benefit. Efforts to accomplish this goal are a major focus of the biomedical enterprise (Fruman et al., 2017).

At the molecular level, PI3K signaling involves the generation of the plasma membrane (PM) second messenger lipids phosphatidylinositol 3,4,5-trisphosphate (PIP_3_) and phosphatidylinositol 3,4-bisphosphate (PI(3,4)P_2_) that activate downstream effector proteins like the serine/threonine kinase Akt. PIP_3_ is the major lipid produced, and most functions of the pathway are attributable to it (Vanhaesebroeck et al., 2012). PI(3,4)P_2_ has instead been viewed as either a degradation product (Ishihara et al., 1999) or an alternative activator of the pathway (Ebner et al., 2017). However, selective functions for PI(3,4)P_2_ have recently been described that are independent of PIP_3_ (Li and Marshall, 2015). These include the formation of lamellipodia and invadopodia (Krause et al., 2004; Oikawa et al., 2008; Bae et al., 2010; Sharma et al., 2013), along with clathrin-mediated and clathrin-independent endocytosis (Posor et al., 2013; Boucrot et al., 2015). In each case, these functions could conceivably be driven by, or occur independently of, class I PI3K signaling.

Synthesis of PI(3,4)P_2_ can proceed via three routes. In the first, class I PI3K directly generates PI(3,4)P_2_ and PIP_3_ by 3-OH phosphorylation of the respective PM phosphoinositides PI4P and PI(4,5)P_2_ (Carpenter et al., 1990). Subsequently, the observation that PI(3,4)P_2_ synthesis lags behind PIP_3_ in stimulated cells (Stephens et al., 1991; Hawkins et al., 1992; Jackson et al., 1992), coupled with the discovery of the PIP_3_-specific 5-phosphatase enzymes SHIP1 and SHIP2 (Damen et al., 1996; Pesesse et al., 1997), led to the proposal of a second route: PI(3,4)P_2_ production by removal of the 5-OH phosphate from PIP_3_. More recently, a third route has been established, again invoking direct phosphorylation of PI4P, this time by class II PI3K enzymes (Domin et al., 1997; Posor et al., 2013). However, the relative contributions of these pathways, and how they couple to disparate PI(3,4)P_2_-dependent cellular functions, remain unclear (Li and Marshall, 2015).

Resolving how the spatial/temporal dynamics of PI(3,4)P_2_ signaling couples to different biological functions requires approaches to identify the lipid in intact, living cells. Isolated lipid binding domains fused to fluorescent reporters often make highly selective genetically encoded biosensors for this purpose (Wills et al., 2018). The pleckstrin homology (PH) domain on the C terminus of Tandem Ph-domain containing Protein 1 (TAPP1) exhibits specific binding to PI(3,4)P_2_ in the test tube (Dowler et al., 2000; Thomas et al., 2001). As a result, several studies have employed fluorescent protein conjugates of this domain to track PI(3,4)P_2_ signaling, though the domain fails to detect resting levels or the limited accumulation of the lipid in response to stimuli such as insulin-like growth factor (Kimber et al., 2002; Marshall et al., 2002; Oikawa et al., 2008; Posor et al., 2013).

Herein, we developed a higher-avidity tandem trimer of PH-TAPP1. We show PI(3,4)P_2_ generation is sufficient to recruit the probe, which is exquisitely selective for the lipid over other phosphoinositides. We then demonstrate that the class I PI3K pathway, acting via PIP_3_ synthesis, dominates PI(3,4)P_2_ accumulation in cells. Our data also support the recently proposed direct degradation of both PI(3,4)P_2_ and PIP_3_ by the lipid phosphatase and tumor suppressor PTEN (Malek et al., 2017). Collectively, our data show that the class I PI3K pathway is the most potent driver of PI(3,4)P_2_-dependent signaling.

## Results

### C-terminal PH domain (cPH) probes selectively bind PM PI(3,4)P_2_

**Figure 1.**
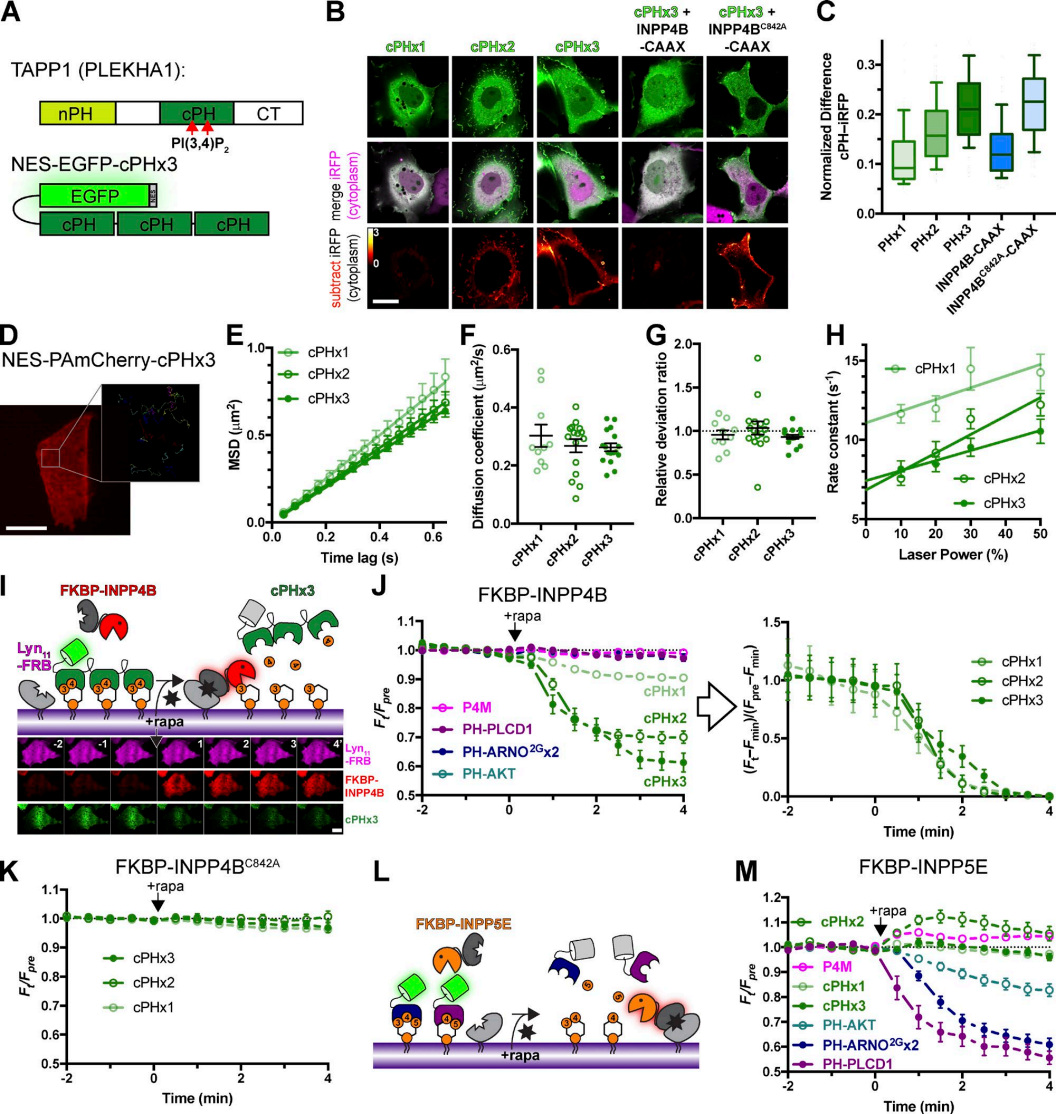
**TAPP1 cPHx3 binds PM PI(3,4)P_2_ selectively. (A)** Domain structure of full-length TAPP1 protein, along with the EGFP-tandem cPHx3 fusion incorporating a nuclear export sequence (NES). **(B and C)** cPHx2 and cPHx3 bind the PM in a PI(3,4)P_2_-dependent manner in COS-7 grown in 10% serum. Confocal sections (B) are shown of cells expressing EGFP-cPH plasmids and iRFP to mark the cytoplasm. Subtracting normalized iRFP signal from normalized EGFP reveals PM localization of cPHx2 and cPHx3, which is removed by coexpressing CAAX-box tagged PI(3,4)P_2_-phosphatase INPP4B but not its inactive mutant C842A. Scale bar is 20 µm. The box and whisker plots show median, interquartile range, and 10th–90th percentiles of 90–93 cells from three independent experiments. **(D–H)** Rapid Brownian diffusion of cPH probes. **(D)** Image shows COS-7 cell expressing PAmCherry-cPHx3 after photoactivation in TIRF. The inset shows prior trajectories of individual molecules activated at low efficiency, imaged at 16.7 Hz. Scale bar is 20 µm (2 µm for inset). **(E)** Mean square displacement versus time lag plots for indicated cPH probes; data are grand means with SEM of 10 (cPHx1), 16 (cPHx2), or 17 (cPHx3) cells. Individual mean cellular diffusion coefficients (F), relative deviation ratio from Brownian motion (G), and off-rate constant of trajectory lifetimes (from one-phase exponential fit at each laser power, 10–11 cells) are shown with means ± SEM. **(I–K)** Acute PI(3,4)P_2_ depletion removes cPH probes from the PM. Chemically induced dimerization between FKBP and FRB to recruit INPP4B to the PM (I). Montage shows TIRF images of a representative COS-7 cell. Scale bar is 20 µm. The graphs in J show normalized fluorescence intensity in TIRF of cells expressing Lyn_11_-FRB-iRFP, mCherry-FKBP-INPP4B, and the indicated EGFP-lipid biosensor. Data are means ± SEM of 28–30 cells from three independent experiments. The graph at right is the same data normalized to the maximum change in fluorescence for each cPH construct, to emphasize similar dissociation kinetics. The graph in K shows the same experiment for cells expressing a catalytically inactive INPP4B; data are means ± SEM of 27–30 cells from three independent experiments. **(L and M)** PIP_3_ or PI(4,5)P_2_ depletion does not deplete cPH from the PM. The same experiment as in I is depicted, except 5-phosphatase FKBP-INPP5E replaces INPP4B. The graph in M shows mean ± SEM of 28–30 cells from three independent experiments.

The TAPP1 protein (encoded by the *PLEKHA1* gene) contain both N-terminal PH domains and cPHs (Fig. 1 A), the latter of which selectively binds PI(3,4)P_2_ (Dowler et al., 2000). Previous studies using the isolated TAPP1 cPH as a lipid biosensor found no detectable translocation in response to stimuli that induce modest PI(3,4)P_2_ accumulation (Kimber et al., 2002). To improve avidity, tandem dimers of TAPP1 fragments containing cPH have been used (Oikawa et al., 2008; Posor et al., 2013; He et al., 2017). However, these constructs are based on a fragment including the entire C terminus (Furutani et al., 2006), which carries binding sites for other proteins in its tail, and have been shown to induce dominant negative effects (Kimber et al., 2002; Hogan et al., 2004; Thalappilly et al., 2008; Li and Marshall, 2015). Therefore, we built tandem dimers and trimers of the isolated cPH (residues 169–329 in human TAPP1) as previously defined (Marshall et al., 2002) and included a nuclear export sequence (Fig. 1 A).

Expression of EGFP-tagged cPH monomers, dimers, or trimers in COS-7 cells maintained in 10% serum exhibit a predominantly cytosolic localization of the probe when viewed by confocal microscopy (Fig. 1 B). However, comparison with another cytosolic protein (infrared fluorescent protein [iRFP]) revealed enrichment of cPHx2 and cPHx3 at the cell periphery; normalization of the two signals followed by subtracting the iRFP signal from that of EGFP (Materials and methods section) showed striking cPHx2/3 peripheral localization (Fig. 1 B). This signal was abolished by a PM-targeted INPP4B phosphatase that specifically degrades PI(3,4)P_2_ (Gewinner et al., 2009), though not by a catalytically inactive mutant (Fig. 1 B). Quantification of the difference between EGFP and iRFP signals (Fig. 1 C with Kruskal–Wallis statistic 152.5; P < 10^−4^) yielded significantly increasing signal from cPHx1 to cPHx3; INPP4B-CAAX significantly reduced cPHx3 signal (P < 10^−4^ in each case; Dunn’s multiple comparison test), whereas the C842A inactive mutant did not (P > 0.99). Therefore, cPHx2 and cPHx3 could detect PI(3,4)P_2_ at the PM of living cells in the presence of serum.

A potential caveat to using tandem arrays of lipid binding domains is that the resulting probe may cluster lipids and exhibit aberrant localization or mobility in the membrane. To determine whether this was the case, we performed single-molecule imaging of cPHx1, cPHx2, and cPHx3 mobility in the PM by tagging with photoactivatable mCherry (PAmCherry). As shown in Fig. 1 D, full activation of the probe with violet light led to uniform labeling of the PM in total internal reflection fluorescence (TIRF) microscopy (TIRFM), whereas low activation intensities allowed us to image single molecule trajectories (Manley et al., 2008). Analysis of the mean square displacement of these trajectories over time revealed free Brownian motion of all three probes (Fig. 1 E). cPHx1, cPHx2, and cPHx3 diffused with an apparent diffusion coefficient of ∼0.3 µm^2^/s (Fig. 1 F) and did not substantially differ (F = 0.71, P = 0.50, one-way ANOVA), and all exhibited a relative deviation ratio close to 1 (Fig. 1 G), meaning there was no substantial difference in actual displacement relative to that predicted by free Brownian diffusion (Fujiwara et al., 2016). Therefore, the tandem array of domains did not slow the probe’s mobility in the membrane.

The lifetime distribution of the single molecules on the membrane followed a single exponential decay with a characteristic off-rate constant (*k*_off_) that increased linearly with laser power due to increased photobleaching (Fig. 1 H). Extrapolation of this off rate to the intercept at zero illumination power allowed us to estimate the true off rate of the probes. cPHx1 has a lifetime on the membrane of ∼90 ms (off rate = 11 s^–1^), whereas cPHx2 and cPHx3 both showed lifetimes of ∼140 ms. This change in dissociation rate of the tandem arrays was comparatively modest and certainly not multiplicative as would be expected from multiligand binding. This implies that for the majority of probe molecules at steady state, the PH domains are not all occupied by lipid, most likely because of the low abundance of PI(3,4)P_2_. The enhanced PM binding observed in Fig. 1 C likely stems from transient dual or triple occupancy of the PH domains slowing the overall off rate of the population (and perhaps enhancing the on rate for cPHx3 vs. cPHx2).

A potential limitation of biosensors is that when bound to lipid, they will protect the lipid from consumption by enzymes and occlude interaction with endogenous effector proteins, both of which may be needed for local enrichment of the lipid. Therefore, free diffusion of the probe:lipid complex can disrupt local enrichment rather than reporting on it. From the relationship r = (2*D*/*k*_off_)^0.5^ (Teruel and Meyer, 2000), the distance r that cPHx3 typically diffuses while attached to lipid can be estimated as ∼290 nm, not much larger than the diffraction limit. This implies there will be a limited propensity of the probes’ free diffusion to “smear out” local enrichment of PI(3,4)P_2_ molecules that are detectable with diffraction-limited optical imaging.

To determine if PM localization of cPH probes is sensitive to acute depletion of PI(3,4)P_2_, we used chemically induced dimerization between FK506 binding protein 12 (FKBP) and the FKBP12 Rapamycin Binding (FRB) domain of mTor (Belshaw et al., 1996) to recruit INPP4B to the PM (Fig. 1 I). Rapamycin-induced dimerization and thus INPP4B recruitment led to depletion of PM-associated cPH signal in TIRFM (Fig. 1 J; two-way ANOVA of area under the curve before and after rapamycin addition, *F*(1, 200) = 314.5, P < 10^−4^; P < 10^−4^ for cPHx1, cPHx2, and cPHx3, Sidak’s multiple comparisons test). The depletion was progressively greater for cPHx3 > cPHx2 > cPHx1 (comparing area under the curve, *F* = 75.64; P < 10^−4^; cPHx2 vs. cPHx3, P = 0.02; cPHx1 vs. cPHx2, P < 10^−4^), reflecting the increased avidity of the tandem probes for PM PI(3,4)P_2_. On the other hand, probes for other inositol lipids, including PI4P (P4Mx1), PI(4,5)P_2_ (PH-PLCD1), PIP_3_ (PH-ARNO^2G^x2), or PI(3,4)P_2_/PIP_3_ (PH-AKT), did not show a significant change (Várnai and Balla, 1998; Venkateswarlu et al., 1998; Watton and Downward, 1999; Hammond et al., 2014). Notably, when the data were normalized to emphasize the kinetics of the change, no difference between the rates of cPHx1, cPHx2, or cPHx3 dissociation after INPP4B recruitment was observed (Fig. 1 J, right; Kruskal–Wallis statistic = 1.19, P = 0.55), indicating that the higher-avidity tandem probes did not effectively sequester lipid from the INPP4B enzyme. Furthermore, recruitment of inactive INPP4B mutant C842A did not lead to substantial depletion of the cPH probes from the PM (Fig. 1 K).

Given the much higher avidity of cPHx3 and cPHx2 for the PM, we wanted to rule out a weak interaction with other lipids that might synergize to influence PM targeting by these probes. To this end, we used chemically induced dimerization again, this time using the INPP5E phosphatase domain (Fig. 1 L), since this domain is active against PI(4,5)P_2_ and PIP_3_, generating PI4P and PI(3,4)P_2_, respectively (Asano et al., 1999; Kong et al., 2000). INPP5E recruitment did not displace PI4P-binding P4Mx1 or any of the cPH probes from the PM, though it did displace probes that bound to PIP_3_ or PI(4,5)P_2_ (Fig. 1 M). Therefore, these two inositol lipids do not influence the PM association of cPH probes.

Previous studies have implicated a role for PI(3,4)P_2_ at clathrin-coated structures (Posor et al., 2013; He et al., 2017). We tested for an enrichment for our cPHx3 probe at these structures in 293A cells in which the endogenous clathrin had been labeled with a split GFP approach (Leonetti et al., 2016); mCherry-cPHx3 was not enriched at these structures, though an mCherry-tagged PI3K-C2α construct clearly was (Fig. S1 A). This is not surprising, since (1) another inositol lipid firmly established in clathrin-mediated endocytosis, PI(4,5)P_2_, is also not seen to be enriched at clathrin-coated structures (He et al., 2017) and (2) careful modeling of the quantities of PI(3,4)P_2_ required for bud maturation posits that there may be just enough to interact with the endogenous SNX9 effector protein (Schöneberg et al., 2017), resulting in undetectable biosensor recruitment.

A recent strategy found that addition of the clathrin binding module from Auxilin1 generates a coincidence detector for inositol lipids at clathrin-coated structures (He et al., 2017). With such coincident detector probes, we could measure PI(4,5)P_2_ enrichment with a PH-PLCδ1 but not with our cPHx3 PI(3,4)P_2_ probe (Fig. S1 A). This was in contrast with a tandem C-terminal truncation, also from human TAPP1, which does show enrichment in previous reports (He et al., 2017) and in our experiments (Fig. S1 A). What is the cause of this discrepancy? We noted that the C terminus of TAPP1 contains a Leu-Val-Asp-Leu-Asp clathrin binding box that could explain enrichment at clathrin-coated structures. Indeed, a tandem dimer of the C-terminal domain without the PI(3,4)P_2_-binding PH domain still enriched at clathrin-coated structures when fused to the Auxilin1 clathrin binding module (Fig. S1 A). In support of these findings, we found that the TAPP1 PH-domain + C-terminal tandem dimer fused to the Auxilin1 module did not dissociate from the membrane after INPP4B recruitment, when cPHx3 does (Fig. S1 B). Therefore, enrichment of TAPP1-derived protein fragments at clathrin-coated structures does not depend on PI(3,4)P_2_.

Together, these results allowed us to conclude that (1) the cPHx3 probe derived from TAPP1 exhibits more prominent membrane localization than previously used single and tandem dimer versions, (2) the tandem trimer configuration does not disrupt free diffusion in the plane of the membrane nor sequester PI(3,4)P_2_ to an extent that prevents INPP4B access and (3) the tandem trimer configuration exhibits localization that depends solely on the phosphoinositide PI(3,4)P_2_. These are crucial criteria in the definition of a reliable genetically encoded lipid biosensor. However, there is another critical criterion that must be met, which we tested next.

### PI(3,4)P_2_ is sufficient for cPHx3 cellular localization

We have argued that a crucial but often ignored criterion for a high-fidelity lipid biosensor is a sole requirement of the lipid to drive biosensor localization (Wills et al., 2018). A convenient way to test this criterion is to induce ectopic synthesis of the lipid elsewhere in the cell and test whether this is sufficient to recruit the biosensor (Hammond et al., 2014). To this end, we turned to the *Legionella* effector protein LepB, the N terminus of which possesses PI3P 4-OH kinase activity, thus generating PI(3,4)P_2_ via a noncanonical pathway not used by mammalian cells (Dong et al., 2016). We found that expression of LepB led to substantial PI3P depletion from cells and consequent swelling of the endosomal compartment that depends on this lipid for function (unpublished data). Therefore, to induce acute LepB activity, we turned to an optogenetic approach that utilizes genetic code expansion to incorporate an unnatural, caged amino acid into the protein (Luo et al., 2014; Liu et al., 2017; Courtney and Deiters, 2018).

**Figure 2.**
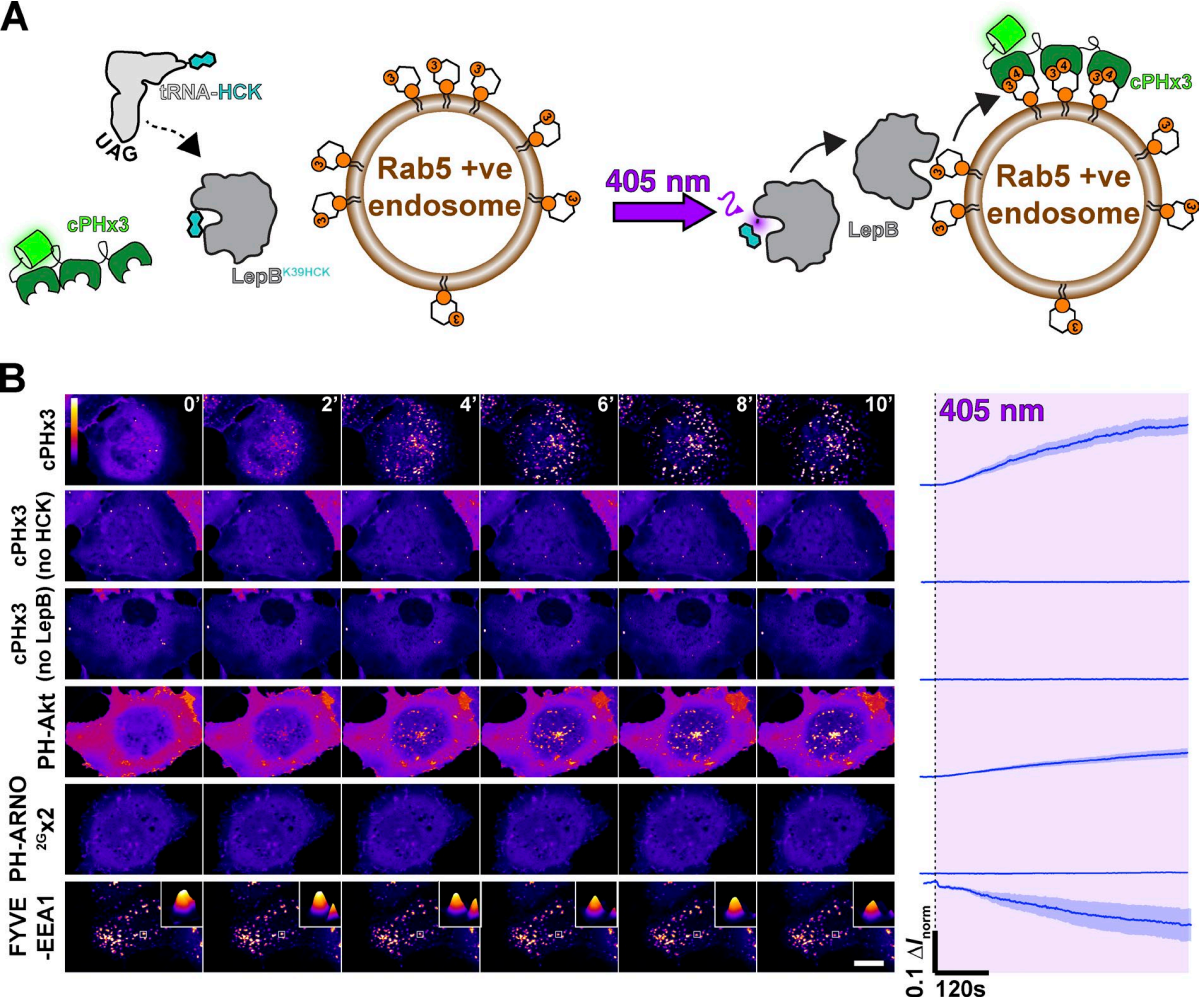
**PI(3,4)P_2_ is sufficient to recruit cPHx3. (A)** Optogenetic activation of PI3P 4-kinase LepB. Cells are transfected with a plasmid encoding Amber stop codon (UAG) recognizing tRNA and a tRNA synthase incorporating hydroxycumarin lysine (HCK), along with a LepB mutant containing a K39 to UAG mutation, causing incorporation of active site blocking HCK into the protein. Upon illumination with 405-nm light, the hydroxycumarin is photolyzed, yielding wild-type K39 and inducing PI(3,4)P_2_ synthesis on PI3P-replete endosomes. **(B)** LepB synthesis of PI(3,4)P_2_ on endosomes recruits cPHx3 and PH-Akt but not the PIP_3_ biosensor PH-ARNO^2G^x2. Images show confocal sections of COS-7 cells transfected with the indicated EGFP-tagged biosensor and the components described in A and grown in the presence of HCK. Scale bar is 20 µm. Two controls are shown where either HCK or LepB plasmids were omitted. The graph shows the change in normalized fluorescence intensity in a mask derived from Rab5 puncta (not shown). Trace lines represents mean, and shaded area is SEM of 19 (cPHx3), 18 (PH-Akt), or 17 (FYVE and PH-ARNO^2G^x2) cells from four independent experiments or 5 cells (no HCK or no LepB) from a single experiment.

This system relies on mutating the target protein at the desired codon to the infrequently used amber stop codon (UAG). The mutant gene is then transfected into cells along with plasmids encoding an engineered pyrrolysyl-tRNA synthetase/tRNA pair and the caged amino acid is added to the media. Here a hydroxycoumarin-caged lysine (HCK) is transacylated onto the tRNA and thence ribosomally incorporated into the mutated gene in response to the UAG codon. In the context of LepB, the bulky hydroxycoumarin group incorporated onto lysine 39 blocks the active site (Dong et al., 2016). However, illumination of cells expressing this system with 405-nm light causes photolysis of the coumarin group, liberating the lysine residue and generating wild-type, active LepB (Fig. 2 A). This provides precise and acute spatiotemporal control over LepB function in live human cells.

Optogenetic activation of LepB in cells expressing cPHx3 caused synthesis of PI(3,4)P_2_ on endosomes and striking recruitment of the cPHx3 probe (Fig. 2 B). This optogenetic activation was only observed in cells grown in the presence of exogenous HCK and depended on cotransfection with the LepB^K39UAG^, ruling out off-target effects on endogenous UAG-containing genes or of light exposure alone. Similarly, we observed clear translocation of PH-AKT to endosomes, which is expected given this PH domain’s binding to both PIP_3_ and PI(3,4)P_2_ (Ebner et al., 2017). However, no recruitment of the PIP_3_-specific PH-ARNO^2G^x2 was observed (Venkateswarlu et al., 1998; Manna et al., 2007), demonstrating that there was no further phosphorylation of the lipid at the 5-OH. Finally, we observed some depletion of the PI3P biosensor FYVE-EEA1 (Wills et al., 2018), though the biosensor remained mostly endosome bound, indicating a small fraction of total PI3P was converted to PI(3,4)P_2_ over the time course of these experiments (Fig. 2 B).

Collectively, these results demonstrate that generation of PI(3,4)P_2_ in a cellular membrane is indeed sufficient to recruit cPHx3, which, given its extensive in vitro selectivity (Dowler et al., 2000; Thomas et al., 2001), now passes all the key criteria that define a high-fidelity, genetically encoded biosensor for PI(3,4)P_2_ (Wills et al., 2018). The fidelity of the probe established, we next turned our attention to deploying this tool to answer some central questions about the lipid’s metabolism and function.

### Dominance of class I PI3K in PI(3,4)P_2_ synthesis

The canonical view of PI(3,4)P_2_ synthesis induced by class I PI3K signaling is that PI3K generates PIP_3_, which is then dephosphorylated at the 5-OH position by inositol polyphosphatase 5-phosphatase (INPP5) family members SHIP1 or SHIP2 (Vanhaesebroeck et al., 2012). Both enzymes are activated by tyrosine phosphorylation cascades initiated by the activated receptor tyrosine kinase. The extent of PI(3,4)P_2_ accumulation varies depending on receptor activation, with insulin or insulin-like growth factor reported to generate only modest amounts of the lipid (Jackson et al., 1992; Guilherme et al., 1996), which have not been detected with a single cPH probe (Kimber et al., 2002).

**Figure 3.**
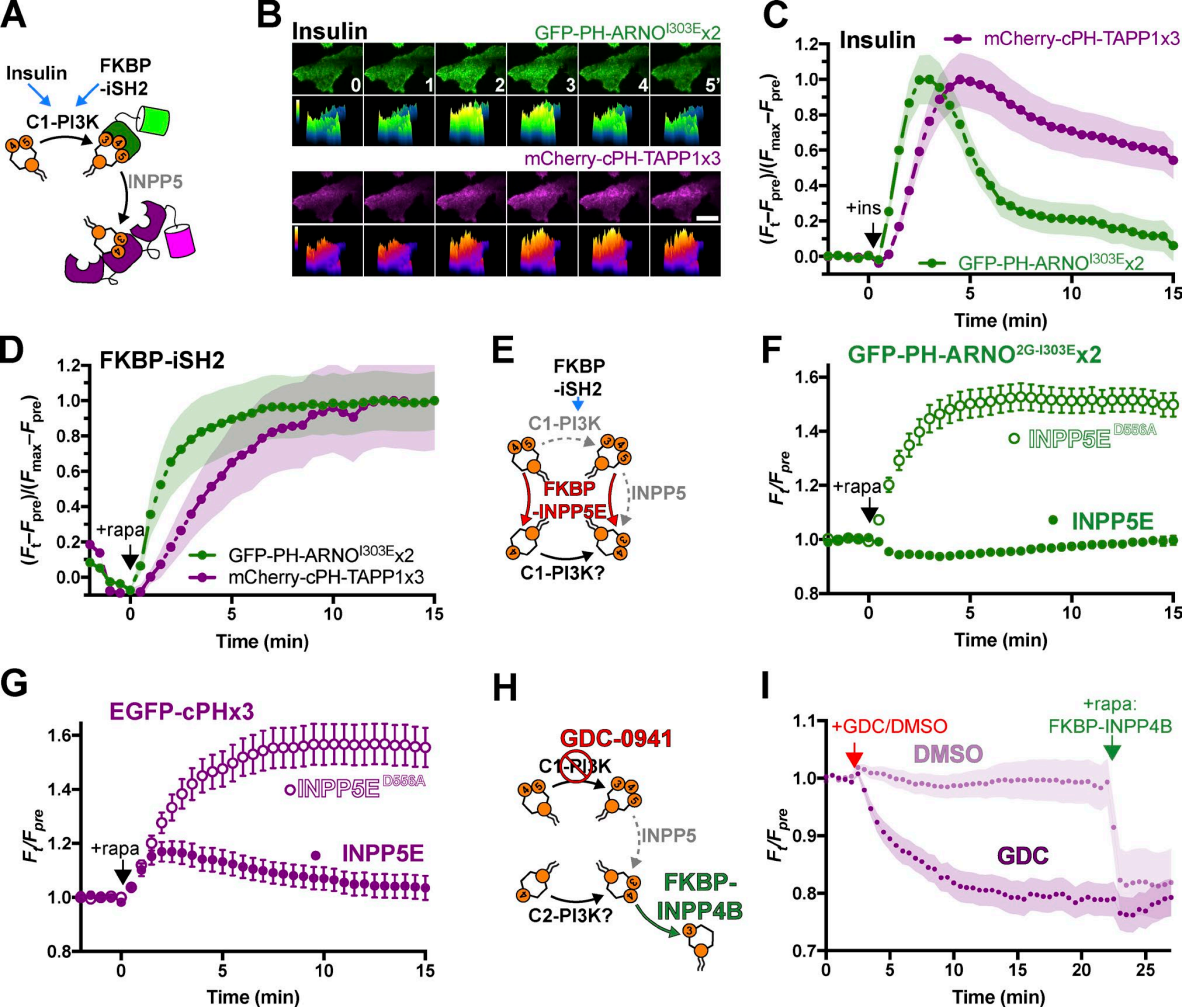
**PM PI(3,4)P_2_ is derived from PIP_3_ synthesized via the class I PI3K pathway. (A)** Activation of the class I PI3K (C1-PI3K) pathway via insulin stimulation or recruitment of an endogenous p110 class I PI3K subunit via chemically induced dimerization–mediated recruitment of FKBP-iSH2. **(B and C)** Insulin-stimulated transient synthesis of PIP_3_ and sustained lagging synthesis of PI(3,4)P_2_. TIRF images of HeLa cells (B) expressing PIP_3_ biosensor EGFP-PH-ARNO^2G-I303E^x2 and PI(3,4)P_2_ biosensor mCherry-cPHx3 stimulated after time 0 with 200 nM insulin. Scale bar is 20 µm. The graph in C shows fluorescence intensity data normalized to minimum and maximum intensities. Data are means with SEM shaded for 34 cells from three independent experiments. **(D)** Artificial activation of class I PI3K with iSH2 also induced lagging synthesis of PI(3,4)P_2_. Data are from COS-7 cells expressing the indicated biosensors, along with Lyn_11_-FRB-iRFP and TagBFP2-FKBP-iSH2. They are normalized to minimum and maximum intensities and are means with SEM shaded for 31 cells from three independent experiments. **(E–G)** PI(3,4)P_2_ is derived from PIP_3_. **(E)** The experimental setup is to activate class I PI3K with FKBP-iSH2 while simultaneously recruiting the PIP_3_ and PI(4,5)P_2_-depleting enzyme FKBP-INPP5E or catalytically inactive D556A mutant as control. Depletion of the PI3K substrate PI(4,5)P_2_ leaves 3-phosphorylation of PI4P as the only route of PI(3,4)P_2_ synthesis. Graphs show data from COS-7 cells imaged by TIRF expressing PIP_3_ biosensor EGFP-PH-ARNO^2G-I303E^x2 (F) or PI(3,4)P_2_ biosensors cPHx3 (G) along with Lyn_11_-FRB-iRFP, TagBFP2-FKBP-iSH2, and mCherry-FKBP-INPP5E wild-type or D556A, as indicated. Data are means ± SEM of 32–40 cells from three independent experiments. **(H and I)** Most PI(3,4)P_2_ is derived from class I PI3K. **(H)** The specific inhibitor GDC-0941 can distinguish class I from class II PI3K activity that could directly produce PI(3,4)P_2_. **(I)** The graph shows normalized fluorescence intensity EGFP-cPHx3 in cells cotransfected with Lyn_11_-FRB-iRFP and mCherry-FKBP-INPP4B, with chemically induced dimerization at 22 min to deplete remaining PI(3,4)P_2_. 250 nM GDC-0941 or DMSO was added at 2 min. Data are means with SEM shaded for 34 cells from four independent experiments (GDC) or 18 cells from two independent experiments (DMSO).

We tested the capacity of cPHx3 to detect insulin-induced PI(3,4)P_2_ production in HeLa cells (Fig. 3 A). To unambiguously detect PIP_3_ in the same cells, we used a tandem dimer of the PH domain from ARNO, specifically selecting the 2G splice variant with high PIP_3_ affinity (Cronin et al., 2004) and introducing the I303E mutation that prevents PH domain interaction with Arl GTPases (Hofmann et al., 2007). We selected this domain since it exhibits clear discrimination for PIP_3_ over PI(3,4)P_2_ (Manna et al., 2007). Insulin stimulation of HeLa cells generated a robust, transient increase in PIP_3_ biosensor at the PM, with a lagging, yet more sustained appearance of the PI(3,4)P_2_ biosensor (Fig. 3, B and C)—a clear recapitulation of the original biochemical measurements of PI3K product generation (Stephens et al., 1991; Hawkins et al., 1992; Jackson et al., 1992), apparently supporting the canonical view.

As an alternative mechanism to activate class I PI3K, we turned to chemically induced dimerization to recruit the inter-Src homology 2 (iSH2) domain from the p85 regulatory subunit of PI3K (Fig. 3 A). This system recruits endogenous PI3K p110 catalytic subunits to the membrane, inducing PIP_3_ synthesis (Suh et al., 2006). However, in this situation, tyrosine kinase-mediated activation of SHIP phosphatases is not expected. Nevertheless, iSH2 recruitment to the PM of COS-7 cells induced rapid PIP_3_ synthesis and the same, lagging PI(3,4)P_2_ accumulation (Fig. 3 D). Use of the selective inhibitor GDC-0941 confirmed this was due to class I-PI3K (Fig. S2 A). We therefore speculated that, in this context at least, PI(3,4)P_2_ accumulation might be driven by direct phosphorylation of PI4P by p110, given that the enzyme is known to perform this reaction in a test tube (Carpenter et al., 1990).

To test this speculation, we devised an experiment wherein the INPP5E enzyme would be corecruited to the PM in conjunction with iSH2. Since INPP5E will deplete both PI(4,5)P_2_ and PIP_3_, accumulation of PIP_3_ should be prevented (Fig. 3 E). PIP_3_ will be degraded into PI(3,4)P_2_, but synthesis of PI(3,4)P_2_ will only be sustained if the p110 enzymes indeed directly convert PI4P into PI(3,4)P_2_. Compared with controls using a catalytically impaired INPP5E D556A mutant, INPP5E completely blocked the accumulation of the PIP_3_ biosensor at the PM after corecruitment (Fig. 3 F). However, cPHx3 initially recruited to the PM at a similar rate to control, though its synthesis was rapidly cut off when INPP5E was corecruited (Fig. 3 G). Therefore, PI(3,4)P_2_ synthesis cannot be sustained in the absence of PIP_3_ generation, even under conditions where SHIP phosphatases are not predicted to become activated. Presumably, the PIP_3_ 5-phosphatase activity responsible comes either from basal SHIP activity through interaction of its C2 domain with acidic PM lipids (Ong et al., 2007; Le Coq et al., 2017) or else from other INPP5 family members, which are all competent at converting PIP_3_ to PI(3,4)P_2_ (Trésaugues et al., 2014). Identification of the enzymes responsible will be a key question for future work. We also noted that the increase in PI(3,4)P_2_ after combined iSH2 and INPP5E recruitment was transient. This is most likely due to endogenous INPP4 or PTEN (see Direct hydrolysis of PI(3,4)P_2_ by PTEN section) degrading PI(3,4)P_2_. The decline could be prevented by an inhibitor, bpV(phen) (bisperoxovanadium 1,10-phenanthroline), that blocks both of these enzymes (Spinelli et al., 2015), as shown in Fig. S2 B.

Thus far, these experiments addressed the pool of PI(3,4)P_2_ generated downstream of class I PI3K signaling. However, activity of class II PI3K has been shown to function in a more constitutive capacity (Posor et al., 2013; Marat et al., 2017). We therefore wanted to identify the source of PI(3,4)P_2_ that we observed at the PM in growing cells (Fig. 1). To this end, we employed the potent and class I PI3K-selective inhibitor GDC-0941 (Kong et al., 2010) at 250 nM to distinguish class I and class II activities (Fig. 3 H). After inhibitor treatment for 20 min, cells were subject to chemically induced dimerization to recruit a coexpressed FKBP-INPP4B to the PM (Fig. 3 I), the goal being to degrade any remaining PI(3,4)P_2_. As shown in Fig. 3 I, GDC treatment led to the depletion of almost the entire pool of PM-associated PI(3,4)P_2_ from cells grown in serum. We cannot rule out that the small additional decrease induced in the 5 min after rapamycin addition is not an addition artifact (Fig. 3 I); area under the curve measurements for after rapamycin treatment versus the 5-min period preceding it do not show a significant change (P = 0.43, Sidak’s multiple comparisons test) as compared with the change following DMSO treatment (P < 10^−4^, Sidak’s multiple comparison test after two-way repeated-measures test, *F* = 61.9, P < 10^−4^). However, the data do clearly indicate that the overwhelming majority of PI(3,4)P_2_ present in the PM of cells is generated by the class I PI3K pathway.

The decline in PI(3,4)P_2_ after GDC-0941 treatment (Fig. 3 I) began immediately after treatment with the compound and is to be expected since synthesis of its immediate precursor, PIP_3_, is being blocked. However, this decline was already apparent within 4 min, whereas no decline (and even a small increase) was apparent after PIP_3_ was depleted via recruitment of the phosphatase INPP5E in Fig. 1 M. How can this be so? Unlike GDC treatment, INPP5E leads to direct conversion of PIP_3_ into PI(3,4)P_2_, inducing an initial burst of PI(3,4)P_2_ synthesis as all remaining PIP_3_ is converted. Indeed, repeating these experiments in the presence of our PIP_3_ biosensor revealed that initial increases in cPHx3 at the PM mirrored the declines in PIP_3_ (Fig. S2 C, inset). However, imaging for extended period revealed that once PIP_3_ had been depleted, levels of PI(3,4)P_2_ began to decline (Fig. S2 C)—much as they did after recruitment of INPP5E in conjunction with iSH2 in Fig. 3 G. We did note that cPHx3 at the PM did not decline under these conditions as much as it did with GDC-0941 treatment. Possible explanations include (1) the continued activity of PIP5K and CI-PI3K after INPP5E recruitment, permitting greatly reduced but not completely blocked PI(4,5)P_2_, PIP_3_, and PI(3,4)P_2_ synthesis, and (2) the lack of PI(4,5)P_2_ at the PM yielding reduced PTEN activity (Campbell et al., 2003).

### Direct hydrolysis of PI(3,4)P_2_ by PTEN

So far, our data support a canonical view of class I PI3K signaling, which is dominated by conversion of PI(4,5)P_2_ to PIP_3_, followed by degradation back to PI(4,5)P_2_ by PTEN or conversion to PI(3,4)P_2_ via INPP5 enzymes, most prominently SHIP1 and SHIP2 (Vanhaesebroeck et al., 2012; Fruman et al., 2017). PTEN and INPP5 thus represent a bifurcation of the pathway: in the former case, toward a simple inactivation, and in the latter, the conversion to a modified but still active signaling state (Li and Marshall, 2015). PI(3,4)P_2_ is ultimately degraded to PI3P by INPP4A/B, a process intimately linked to endocytosis (Shin et al., 2005). However, it has been perplexing that the accumulation of PI(3,4)P_2_ seen after PI3K activation does not lead to a detectable increase in PI3P levels (Stephens et al., 1991; Jackson et al., 1992), implying an alternative route of degradation. Recently, Malek and colleagues have proposed that PTEN in fact directly converts PI(3,4)P_2_ to PI4P, terminating this signaling as well (Malek et al., 2017). The evidence was based on the requirement for PTEN knockout for EGF-stimulated PI(3,4)P_2_ accumulation, in addition to showing that a PI(3,4)P_2_ 3-phosphatase activity in MCF10 cell cytosol was lost in PTEN nulls. However, direct evidence for hydrolysis of PI(3,4)P_2_ in intact, living cells is still lacking.

**Figure 4.**
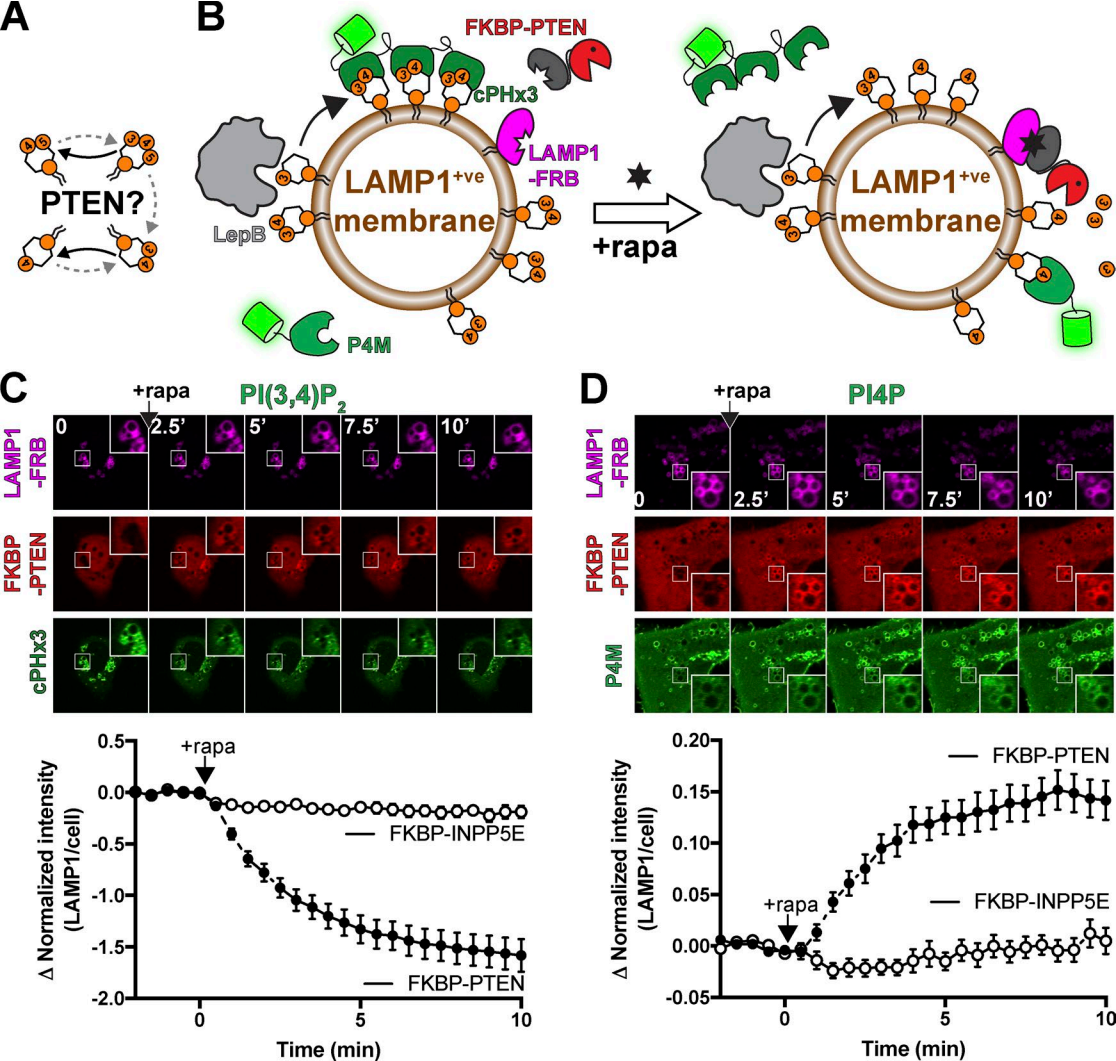
**PTEN directly dephosphorylates PI(3,4)P_2_. (A)** Potential PTEN-driven reactions. **(B)** Experimental setup. COS-7 cells are expressing active LepB to drive PI(3,4)P_2_ in the endosomal system, including (but not limited to) LAMP1-positive membranes. LAMP1-FRB is then used for chemically induced dimerization–mediated recruitment of mCherry-FKBP-PTEN to the membranes, where changes in lipids are detected with the indicated biosensors. **(C and D)** PTEN-mediated conversion of PI(3,4)P_2_ to PI4P. Images show confocal sections of COS-7 cells expressing the indicated constructs before and at the indicated times after chemically induced dimerization with rapa. Insets are 5 µm. Data in the graphs are means ± SEM of 32–37 cells from three independent experiments. FKBP-INPP5E serves as a negative control.

The principle reason for the ongoing ambiguity over cellular activity of PTEN against PI(3,4)P_2_ has been the ambiguity when interpreting changes in lipid levels in cells. When PTEN loss induces PI(3,4)P_2_ accumulation, this can be explained by failure of PTEN to degrade PI(3,4)P_2_ directly—or alternatively, due to impaired PIP_3_ degradation, leaving more substrate available for the INPP5 enzymes (Fig. 4 A). Careful mathematical modeling suggested the latter explanation did not explain the accumulation in MCF10 cells (Malek et al., 2017). Nonetheless, the evidence is indirect. We therefore devised an experiment using the LepB PI3P 4-OH kinase. Since we showed that this enzyme generates an endosomal pool of PI(3,4)P_2_ devoid of PIP_3_ (Fig. 2), activity of PTEN leading to depletion of PI(3,4)P_2_ can be unambiguously assigned to direct hydrolysis of the lipid in this context (Fig. 4 B).

Indeed, use of chemically induced dimerization of FKBP-PTEN to FRB-LAMP1–positive endosomes/lysosomes caused a rapid depletion of endosomal PI(3,4)P_2_ detected with cPHx3 in LepB-expressing COS-7 cells (Fig. 4 C). That this was truly PI(3,4)P_2_ to PI4P conversion induced by PTEN was further indicated by a clear accumulation of PI4P on these membranes (Fig. 4 D). Therefore, our data provide a direct demonstration of PI(3,4)P_2_ hydrolysis by PTEN in intact, living cells and confirm the novel role of this enzyme in terminating PI(3,4)P_2_ signaling in addition to signals driven by PIP_3_ (Malek et al., 2017).

## Discussion

In this study, we report a high-avidity probe for PI(3,4)P_2_, cPHx3-TAPP1, which satisfies three crucial criteria for a genetically encoded lipid biosensor (Wills et al., 2018): (1) binding of the PH domain is exquisitely specific for PI(3,4)P_2_ (Dowler et al., 2000; Thomas et al., 2001; Manna et al., 2007), (2) PI(3,4)P_2_ binding is absolutely necessary, and (3) PI(3,4)P_2_ binding is sufficient for localization in living cells (Figs. 1 and 2). We use this high-fidelity and high-sensitivity probe to develop the first direct evidence for the canonical pathway of PI(3,4)P_2_ synthesis by class I PI3K (i.e., by PIP_3_ dephosphorylation; Fig. 3), first proposed nearly three decades ago (Stephens et al., 1991; Hawkins et al., 1992). We also provide direct evidence for a much more recent proposition (Malek et al., 2017): that the tumor suppressor PTEN also terminates PI(3,4)P_2_ signals in addition to those mediated by PIP_3_ (Fig. 4). We also generated the first optogenetically activatable lipid kinase, LepB (Fig. 2).

How PI(3,4)P_2_ signaling couples to different functions, and their relationship (or not) with the class I PI3K pathway, is still poorly understood (Li and Marshall, 2015; Hawkins and Stephens, 2016). This has largely been due to the difficulty in studying PI(3,4)P_2_ in isolation from the other, more abundant phosphoinositides such as PI(4,5)P_2_ and PIP_3_. However, recent advances in mass spectrometry are allowing sensitive detection of this lipid (Malek et al., 2017; Bui et al., 2018). We anticipate that new tools for optical detection of PI(3,4)P_2_ in living cells will complement these approaches and greatly accelerate research in this area.

While this manuscript was in preparation, another TAPP1-derived PI(3,4)P_2_ biosensor was reported, which like ours also excluded the C-terminal portion of the protein (Liu et al., 2018). This probe reported very similar dynamics for PI(3,4)P_2_ relative to PIP_3_ to those reported here, and the study reached the same conclusion as to the role of class I PI3K in synthesis. Although based on a single PH domain rather than the tandem trimer we report, the probe used by Liu et al. (2018) could detect PI(3,4)P_2_ in response to IGFI in NIH-3T3 cells, a feat not accomplished with previous single-domain probes (Kimber et al., 2002). The difference likely stems from the inclusion of a hydrophobic solvatochromic fluor that penetrates the bilayer, favorably altering the partition coefficient of the probe. This fluor allows ratiometric measurement of membrane association, which is conveniently calibrated against liposomal standards for precise measurement of mole percentage. This improved quantitative capacity, though superior to our sensitive but uncalibrated measurement of membrane localization with cPHx3, is less convenient. The solvatochromic fluor must be covalently attached to recombinant protein and introduced to cells via microinjection (or perhaps electroporation). We therefore believe our simple, transfectable plasmid-based biosensor will greatly benefit efforts to dissect the cellular functions of PI(3,4)P_2_.

For now, the clearest demonstration is that most PI(3,4)P_2_ accumulating in cells is derived from INPP5 activity on class I PI3K-synthesized PIP_3_. Without stimulation, resting levels of PI(3,4)P_2_ are extremely low (Stephens et al., 1991; Hawkins et al., 1992), with immunocytochemical estimates suggesting that perhaps 40% of this is generated by the class II PI3K-C2α (Wang et al., 2018). The function of the greatly expanded class I PI3K-driven pool remains an ongoing question. Conversion of PIP_3_ to PI(3,4)P_2_ will terminate signals generated by PIP_3_-selective effectors, maintain signals by more promiscuous effectors (like Akt), and initiate signaling by PI(3,4)P_2_-selective effectors (Hawkins and Stephens, 2016). What is the purpose of this tiered lipid signaling system? A clue comes from perhaps the best characterized PI(3,4)P_2_-selective effector protein: the TAPP proteins from which our probe is derived. Mice harboring point mutations in *Plekha1* and *Plekha2* that disrupt PI(3,4)P_2_-binding of the TAPP1 and TAPP2 cPHs exhibit augmented PI3K signaling through insulin and B cell receptors (Wullschleger et al., 2011; Landego et al., 2012), suggesting a major function of the lipid is in negative feedback of class I PI3K signaling. Interestingly, PI(3,4)P_2_ is also implicated in endocytosis (Shin et al., 2005; Posor et al., 2013; Boucrot et al., 2015). Therefore, it could be possible that PI(3,4)P_2_ down-regulates PI3K signals by triggering internalization of activated receptors.

The extent to which receptor endocytosis effectively down-regulates class I PI3K signaling is currently unclear. PI(3,4)P_2_ has been reported on early endosomes after PDGF stimulation of NIH-3T3 fibroblasts, where it recruits Akt2, suggesting continued PI3K-mTOR signaling (Liu et al., 2018). In contrast, Rab5 was shown to recruit INPP4A and thus remove PI(3,4)P_2_ from nascent endosomes (Shin et al., 2005). Moreover, we failed to observe an endosomal localization of our PI(3,4)P_2_ probe in serum stimulated 293A cells (Fig. S1 A) or after insulin stimulation of HeLa cells (Fig. 3 B). We also found that a previous report of TAPP1-based probe localization to endocytic structures (He et al., 2017) is likely driven by a clathrin box in the C terminus of the protein (Fig. S1 A). It is of course entirely possible that PI3K signaling capacity from endocytic structures depends on the receptor and cell type. Notably, continued PI3K signaling or termination are both compatible with proposed roles for PI3K-C2α–derived PI(3,4)P_2_ in clathrin-coated vesicle maturation (Posor et al., 2013). Termination of endosomal PI3K signals must occur at some point, however, since continued PI3K-Akt-mTOR activation would conflict with PI3K-C2β–mediated mTOR down-regulation at late endosomes (Marat et al., 2017).

A second major finding is that PI(3,4)P_2_ is directly hydrolyzed by PTEN (Malek et al., 2017). This result is surprising since meticulous work with purified protein found much poorer PTEN activity against PI(3,4)P_2_ versus PIP_3_ (McConnachie et al., 2003). The discrepancy likely derives from the very different enzyme–substrate interactions in living cells; indeed, turnover numbers for PTEN in living cells are several orders of magnitude higher than in the test tube (McConnachie et al., 2003; Feng et al., 2014). It therefore seems likely that PTEN regulates PI(3,4)P_2_ signaling in addition to PIP_3_. The recent observations that PTEN reduced signaling at forming clathrin-coated structures and slows their maturation is entirely consistent with a role for PI(3,4)P_2_ during endocytosis (Rosselli-Murai et al., 2018). Finally, since PTEN’s activity on PI(3,4)P_2_ is the direct reversal of the PI4P conversion mediated by class II PI3K and since PTEN has recently been found to localize to endosomal structures (Naguib et al., 2015), PTEN seems poised to regulate PI3K-C2β signaling from late endosomes/lysosomes too (Marat et al., 2017).

In summary, we have developed and validated a sensitive genetically encoded lipid biosensor for PI(3,4)P_2_. We show this lipid accumulates in cells primarily in response to class I PI3K activity, via circuitous dephosphorylation of PIP_3_. We also provide evidence for direct hydrolysis of PI(3,4)P_2_ by PTEN. Collectively, these results provide fresh impetus to dissect the physiological and pathological signaling outcomes driven by PI(3,4)P_2_ and provide a powerful new tool to aid in this endeavor.

## Materials and methods

### Cell culture and transfection

The COS-7 (ATCC CRL-1651) and HeLa (ATCC CCL-2) cells were cultured in DMEM (low glucose; Life Technologies 10567022) supplemented with 10% heat-inactivated fetal bovine serum (Life Technologies 10438–034), 100 units/ml penicillin, 100 µg/ml streptomycin (Life Technologies 15140122), and 1:1,000 chemically defined lipid supplement (Life Technologies 11905031) at 37°C in a humidified atmosphere with 5% CO_2_. They were passaged twice per week by dissociation in TrpLE (Life Technologies 12604039) and diluting 1 in 5. 293A cells with their endogenous *CLTA* alleles tagged with split GFP were generated exactly as described (Leonetti et al., 2016) using a protocol we have described (Zewe et al., 2018). Briefly, Platinum Cas9 (Thermo Fisher) was precomplexed with gRNA and electroporated into 293A^sfGFP1-10^ cells in combination with a single-stranded HDR template (IDT). After recovery, cells were sorted by FACS for GFP-positive cells. They were cultured under identical conditions to the COS-7 and HeLa cells.

For transfection, cells were seeded in 35-mm tissue culture dishes with 20-mm number 1.5 cover glass apertures (CellVis) coated with 5 µg fibronectin (Life Technologies 33016-015). Between 1 and 24 h after seeding, cells were transfected with 1 µg total plasmid DNA precomplexed with 3 µg lipofectamine2000 (Life Technologies 11668019) in 200 µl Opti-MEM (Life Technologies 51985091) according to the manufacturer’s instructions. Cells were imaged 18–26 h after transfection. For unnatural amino acid incorporation, medium was supplemented with 250 µM hydroxycoumarin lysine in parallel with the transfection.

### Plasmids

The pPAmCherry-C1 (Addgene plasmid 31929) was a kind gift of Vladislav Verkhusha (Albert Einstein College of Medicine, New York, NY; Subach et al., 2009). The EGFP (*Aequorea victoria* GFP with F64L and S65T mutations with human codon optimization), mCherry, *Rhodopseudomonas palustris* bacteriophytochrome BphP2-derived near-iRFP variant iRFP713, and *Entacmaea quadricolor* GFP-like protein eqFP578 variant pTagBFP2 were used in the Clontech pEGFP-C1 and N1 backbones as described previously (Zewe et al., 2018). TAPP1 cPH and LepB sequences were obtained as synthetic gblocks (IDT). All plasmids were verified by dideoxy sequencing. Constructs generated in this study are freely distributed through Addgene. Plasmids were constructed using standard restriction cloning or NEB HiFi assembly (New England Biolabs E5520S) or else obtained from the sources indicated in Table 1.

**Table 1.** Plasmids used in this study

**Plasmid**	**Backbone**	**Insert**	**Reference**
NES-EGFP-cPHx1	pNES-EGFP-C1	*X. laevis* map2k1.L(32-44):EGFP:PLEKHA1(169-329)	This study
NES-EGFP-cPHx2	pNES-EGFP-C1	*X. laevis* map2k1.L(32-44):EGFP:PLEKHA1(169-329):GGSGGSGG: PLEKHA1(169-329)	This study
NES-EGFP-cPHx3	pNES-EGFP-C1	*X. laevis* map2k1.L(32-44):EGFP:PLEKHA1(169-329):GGSGGSGG: PLEKHA1(169-329): GGSGGSGG: PLEKHA1(169-329)	This study
TagBFP2-CAAX	pTagBFP2-C1	TagBFP2:HRAS(172-189)	This study
TagBFP2-INPP4B-CAAX	pTagBFP2-C1	TagBFP2:INPP4B:HRAS(172-189)	This study
TagBFP2-INPP4B^C842A^-CAAX	pPAmCherry-C1	TagBFP2:INPP4B(C842A):HRAS(172-189)	This study
NES-PAmCherry-cPHx1	pPAmCherry-C1	PAmCherry:PLEKHA1(169-329)	This study
NES-PAmCherry-cPHx2	pPAmCherry-C1	PAmCherry:PLEKHA1(169-329):GGSGGSGG: PLEKHA1(169-329)	This study
NES-PAmCherry-cPHx3	pPAmCherry-C1	PAmCherry:PLEKHA1(169-329):GGSGGSGG: PLEKHA1(169-329): GGSGGSGG: PLEKHA1(169-329):	This study
NES-mCherry-cPHx3	pNES-mCherry-C1	mCherry:PLEKHA1(169-329):GGSGGSGG: PLEKHA1(169-329): GGSGGSGG: PLEKHA1(169-329)	This study
pNES-iRFP-C1	piRFP-C1	*X. laevis* map2k1.L(32-44):iRFP	This study
Lyn_11_-FRB-iRFP	piRFP-N1	*LYN*(1-11):*MTOR*(2021-2113):iRFP	Hammond et al. (2014)
LAMP1-FRB-iRFP	piRFP-N1	*LAMP1*:*MTOR*(2021-2113):iRFP	This study
mCherry-FKBP-INPP5E	pmCherry-C1	mCherry:*FKBP1A*(3-108):[GGSA]_4_GG:INPP5E(214-644)	Hammond et al. (2014)
mCherry-FKBP-INPP5E^D556A^	pmCherry-C1	mCherry:*FKBP1A*(3-108):[GGSA]_4_GG:INPP5E(214-644)	Hammond et al. (2014)
mCherry-FKBP-INPP4B	pmCherry-C1	mCherry:*FKBP1A*(3-108):[GGSA]_4_GG:INPP4B	This study
mCherry-FKBP-INPP4B^C842A^	pmCherry-C1	mCherry:*FKBP1A*(3-108):[GGSA]_4_GG:INPP4B(C842A)	This study
mCherry-FKBP-PTEN	pmCherry-C1	mCherry:*FKBP1A*(3-108):[GGSA]_4_GG:PTEN	This study
TagBFP2-FKBP-iSH2	pTagBFP2	TagBFP2:AAAGAGGAA:*FKBP1A*(3-108): [GGSA]_4_GG:Mus musculus *Pik3r1*(159-349)	Suh et al. (2006)
NES-EGFP-P4Mx1	pEGFP-C1	*X. laevis map2k1.L*(32-44):EGFP:*L. pneumophila SidM*(546-647)	Zewe et al. (2018)
PH-PLCD1-EGFP	pEGFP-N1	PLCD1v2(1:170):EGFP	Várnai and Balla (1998)
PH-AKT-EGFP	pEGFP-N1	AKT(1-164):EGFP	Servant et al. (2000)
NES-EGFP-PH-ARNO^2G^x2	pEGFP-C1	*X. laevis* map2k1.L(32-44):EGFP:CYTH2(252-399):GGSGGVDM: CYTH2(252-399)	This study
NES-EGFP-PH-ARNO^2G-I303E^x2	pEGFP-C1	*X. laevis* map2k1.L(32-44):EGFP:CYTH2(252-399)(I303E):GGSGGVDM: CYTH2(252-399)(I303E)	This study
EGF-FYVE-EEA1	pEGFP-C1	EGFP:EEA1(1253-1411)	Balla et al. (2000)
pcDNA3-U6H1-X1-HCKRS	pcDNA3	pyrrolysyl-tRNA synthetase/tRNA	Luo et al. (2014)
TagBFP2-LepB^K39TAG^-iRFP	pTagBFP2-C1	TagBFP2: *L. pneumophila LepB^K39TAG^*(1-311):GGSGG:iRFP	This study
mCherry-Rab5	pmCherry-C1	mCherry:*Canis lupus RAB5A*	Hammond et al. (2014)
mCherry-PI3K-C2α	pmCherry-C1	mCherry:PIK3C2A	Posor et al. (2013)
mCherry-cPH-CTx2-Aux1	pmCherry-C1	mCherry:[GGS]_5_:PLEKHA1(180-404):EF:PLEKHA1(180-404):YRYFQAS:*Bos Taurus* DNAJC6(420-814)	He et al. (2017)
pCherry-cPHx3-Aux1	pmCherry-C1	mCherry:PLEKHA1(169-329):GGSGGSGG: PLEKHA1(169-329): GGSGGSGG: PLEKHA1(169-329):RVDGTAEAS: *Bos Taurus* DNAJC6(420-814)	This study
pmCherry-CTx2-Aux1	pmCherry-C1	mCherry:[GGS]_3_: PLEKHA1(329-404):GGSGGSGG: PLEKHA1(329-404):QAS: *Bos Taurus* DNAJC6(420-814)	This study
pmCherry-PH-PLCδ1-Aux1	pmCherry-C1	mCherry:[GGS]_3_:PLCD1v2(2-175):QAS: *Bos Taurus* DNAJC6(420-814)	He et al. (2017)

### Chemicals

Rapamycin (Thermo Fisher BP2963-1) was dissolved in DMSO at 1 mM and stored in aliquots at −20°C; the final concentration used in cells was 1 µM. 4 mg/ml Insulin zinc solution (Thermo Fisher 12585014) was stored in aliquots at −20°C. The GDC-0941 (EMD-Millipore 5.09226.0001) was dissolved in 2 mM DMSO and stored in aliquots at −20°C. Aliquots of hydroxycoumarin lysine (Luo et al., 2014) were stored at −20°C in DMSO at 100 mM.

### Microscopy

Cells were imaged in 1.6 ml FluoroBrite DMEM (Life Technologies A1896702) supplemented with 25 mM Hepes (pH 7.4) 1:1,000 chemically defined lipid supplement with or without 10% heat-inactivated fetal bovine serum. For treatments, 0.4 ml of this medium containing fivefold the final concentration of compound was applied to the dish (or 0.5 ml for a second addition).

Confocal microscopy was performed on a Nikon TiE inverted stand with an A1R resonant scan head and fiber-coupled four-line excitation (Ex) LU-NV laser combiner equipped with 405-, 488-, 561-, and 640-nm lines. 8 or 16 frame averages were used to improve signal to noise. A 100× 1.45 NA plan-apochromatic oil-immersion objective was used throughout. Blue (405 nm Ex and 425–475 nm emission [Em]) and yellow/orange (561 nm Ex and 570–620 nm Em) channels were imaged concurrently, alternating with concurrent imaging of green (488 nm Ex and 500–550 nm Em), far/infrared (640 nm Ex and 663–737 nm Em), and a transmitted light channel for DIC. The hexagonal confocal pinhole was set to 1.2× Airy disc size of the longest wavelength imaged.

For TIRFM, we used a second Nikon TiE microscope fitted with a TIRF illuminator arm fiber coupled to an Oxxius L4C laser combined equipped with 405-, 488-, 561-, and 640-nm lasers. A 100× 1.45 NA plan-apochromatic oil-immersion objective was used to deliver the high angle of incidence illuminating beam and acquire the images by epifluorescence. Images were acquired on a Zyla 5.5 sCMOS camera (Andor) with 2 × 2 binning in rolling shutter mode. Blue (405 nm) and yellow/orange (561 nm Ex) channels were imaged through a dual-pass 420- to 480-nm and 570- to 620-nm filter (Chroma), whereas green (488 nm) and far/infrared (640 nm Ex) used a dual-pass 505- to 550-nm and 650- to 850-nm filter (Chroma).

Optogenetic activation of LepB was performed by confocal microscopy. After acquiring ∼30 s of data with 405 nm illumination set to zero power, transmission was turned up to 20% of the maximum available power from the LU-NV unit.

For single-molecule imaging, PAmCherry was imaged without pixel binning in global shutter mode with 25-ms exposures and 30% illumination power with 561 nm and 0.8% 405 nm for photoactivation from the 100-mW Oxxius lasers. A 16 × 16-µm region of PM was imaged for tracking.

### Image analysis

Images in Nikon nd2 image format were imported into the open access image analysis package Fiji (Schindelin et al., 2012), using the LOCI BioFormats importer (Linkert et al., 2010). A custom written macro was used to combine fields into a single enlarged image for the purposes of image analysis (though never for presentation).

#### Quantifying the difference in intensities between EGFP and iRFP channels (Fig. 1, B and C)

Regions of interest (ROI) were drawn around each cell, and a custom-written macro was used to measure the average pixel intensity in these ROI and then normalize each pixel to this value. The normalized iRFP (cytosolic) image was then subtracted from the normalized EGFP-cPH channel to yield the subtracted image.

#### Intensity changes in specific compartments by confocal imaging (Fig. 2 B and Fig. 4, B and C)

Cells were measured inside ROI. A second image channel (mCherry-Rab5 in Fig. 2 and LAMP1-FRB-iRFP in Fig. 4) was autothresholded and used to generate a mask to measure the normalized pixel intensity of the EGFP channel, as previously described in detail (Zewe et al., 2018).

#### PM intensity changes imaged by TIRFM (Fig. 1, J–M; and Fig. 3)

The ROI corresponding to the footprint of individual cells were defined, and after subtracting camera noise, intensity levels over time were measured. Average pixel intensity in each frame t was normalized to the pretreatment average level *F*_t_/*F*_pre_.

#### Single molecule analysis (Fig. 1, D–H)

Single molecule trajectories were segmented and tracked using the open-source Fiji implementation of the u-track single particle tracking algorithm (Jaqaman et al., 2008). A difference of Gaussians filter was used with a hard threshold and an estimated dimeter of 0.5 µm for the estimated diameter (i.e., an ∼8 × 8-pixel neighborhood) of single molecules. Coordinates of the trajectories were exported as XML files, and mean-square displacement at different time lags (Vrljic et al., 2007) was calculated along with trajectory lifetime distributions using custom written code in Python.

#### Selecting representative images for presentation

Example images were selected based on having the best signal to noise possible, while also having measured values close to the sampled population median, and are always within the central interquartile range.

Data presentation and statistics was performed using Graphpad Prism 7. Data were subject to the D’Agostino and Pearson normality test to select for parametric or nonparametric tests.

### Online supplemental material

Fig. S1 shows that cPHx3 does not label endocytic or endosomal structures. Fig. S2 shows that PM PI(3,4)P_2_ is derived from PIP_3_ synthesized via the class I PI3K pathway (additional evidence).

## Supplementary Material

Supplemental Materials (PDF)
