# Supplementary material for: A high-avidity biosensor reveals plasma membrane PI(3,4)P2 is predominantly a class I PI3K signaling product
Source: J Cell Biol. 2019 Mar 4;218(3):1066–79. doi: 10.1083/jcb.201809026 (PMC6400549; doi:10.1083/jcb.201809026)
Supplement: Supplemental Materials (PDF) [file JCB_201809026_sm.pdf]

Supplemental material

Goulden et al., https://doi.org/10.1083/jcb.201809026

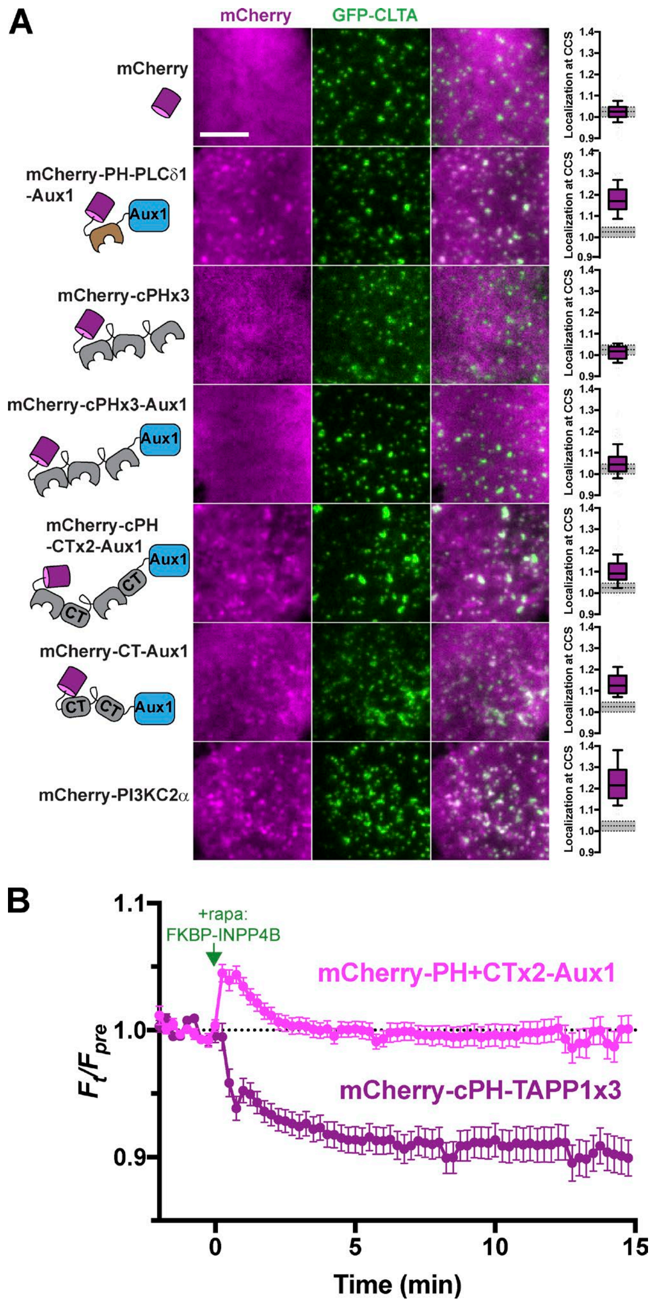

Figure S1. **cPHx3 does not label endocytic or endosomal structures. (A)** The C-terminal domain of TAPP1 is responsible for binding clathrin-coated structures in conjunction with Auxilin1 clathrin binding domain. Images show TIRF of 293A cells with endogenous clathrin tagged with sfGFP in green and the indicated expression construct in magenta. The box and whisker plots show median, interquartile range, and 10th–90th percentiles of 70–151 cells pooled from three or four independent experiments. The shaded regions represent the interquartile range of the mCherry control condition. Scale bar is 5 µm. **(B)** Depletion of PI(3,4)P_2 does not displace mCherry-cPH-CTx2 from the PM. 293A cells with clathrin sfGFP were transfected with the indicated construct along with along with Lyn_{11}-FRB-iRFP and TagBFP2-FKBP-INPP4B. Data are means ± SEM from 49 (cPH-CTx2) or 57 cells (cPHx3) from three independent experiments.

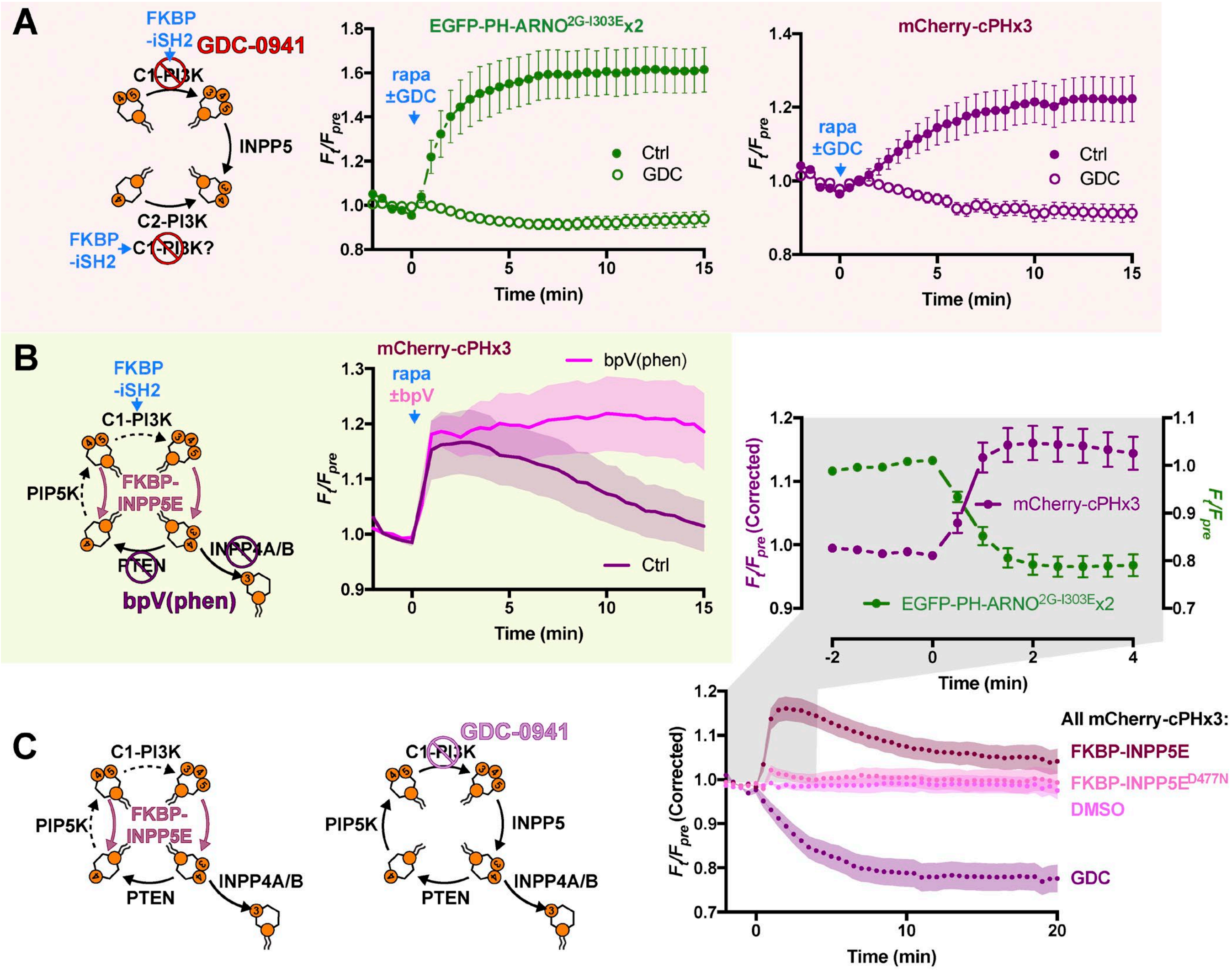

Figure S2. **PM PI(3,4)P_2 is derived from PIP_3 synthesized via the class I PI3K pathway (additional evidence). (A)** iSH2-induced PI(3,4)P_2 synthesis depends on class I PI3K. Data are from COS-7 cells expressing the indicated biosensors, along with Lyn_{11}-FRB-iRFP and TagBFP2-FKBP-iSH2. 1 µM rapamycin ± 250 nM GDC-0941 was added at time 0. Data are normalized to prestimulation average and are means with SEM shaded for 31 (ctrl) or 32 (GDC) cells from three independent experiments. Note that the control data are also plotted after normalizing to minimum and maximum intensities in Fig. 3 D. **(B)** Phosphatase inhibitors prevent PI(3,4)P_2 declines after INPP5E-induced conversion from PIP_3. The COS-7 cells were transfected with Lyn_{11}-FRB-iRFP, FKBP-iSH2 to induce class I PI3K pathway (C1-PI3K) activation, FKBP-INPP5E to deplete PI(4,5)P_2 and PIP_3, as well as EGFP-cPHx3 to report PI(3,4)P_2 levels. At time 0, PM recruitment of the enzymes was induced with 1 µM rapamycin in the presence or absence of 10 µM bpV(phen). Data are means ± SEM of 36 cells from three independent experiments. **(C)** FKBP-INPP5E induces both immediate PI(3,4)P_2 synthesis (from PIP_3) and long-term PI(3,4)P_2 depletion, whereas GDC-0941 induces depletion but not a burst of synthesis. Cells were transfected with FKBP-INPP5E where indicated or the inactive D477N mutant control, along with Lyn_{11}-FRB-iRFP, mCherry-cPHx3, and GFP-PH-$ARNO^{2G-I303E}$x2. They were imaged in the continued presence to 10% serum. At time 0, cells were stimulated with 1 µM rapa (FKBP-expressing cells) or 250 nM GDC-0941 (or 0.025% DMSO as control). Data are means ± SEM of 34–35 cells from three independent experiments. The inset shows the first 4 min of the experiment, illustrating how initial increases in cPHx3 after INPP5E recruitment mirror the depletion of the PIP3 biosensor. Note that in these experiments, we noted substantial photobleaching of the mCherry channel. This was corrected after subtraction of a single exponential fit to the control data, hence the labeling of the cPHx3 data as "corrected." All experiments were imaged with TIRFM.
